# Correction: Resistance mechanism of soluble microbial products to silver nanoparticles in activated sludge: adsorption, bonding and influencing factors

**DOI:** 10.1039/d5ra90106e

**Published:** 2025-09-23

**Authors:** Jia Kang, Ao-di Wang, Yao-wen Zhang, Fei Dai, Jing-Jing Zhu, Chu-qiong Song, Gang-fu Song

**Affiliations:** a School of Environmental and Municipal Engineering, North China University of Water Resources and Electric Power Zhengzhou 450046 China kangjia@ncwu.edu.cn; b Hubei Institute of Water Resources Survey and Design Co., Ltd Wuhan 430070 China; c Henan Urban Planning and Design Institute Co., Ltd Zhengzhou 450044 China

## Abstract

Correction for ‘Resistance mechanism of soluble microbial products to silver nanoparticles in activated sludge: adsorption, bonding and influencing factors’ by Jia Kang *et al.*, *RSC Adv.*, 2025, **15**, 29978–29988, https://doi.org/10.1039/D5RA03336E.

The authors regret the omission of funding information in the Acknowledgements section in the original article. The corrected Acknowledgement is given here.

The Royal Society of Chemistry apologises for these errors and any consequent inconvenience to authors and readers.

